# Feline Kobuvirus in Domestic Cats in Northern Vietnam: Molecular Insights Reveal Adaptive Evolution in VP1 Protein

**DOI:** 10.3390/ijms27167394

**Published:** 2026-08-18

**Authors:** Hieu Van Dong, Giang Thi Huong Tran, Hoang Viet The Nguyen, Linh Ngoc Phan, Amonpun Rattanasrisomporn, Chaiwat Boonkaewwan, Dao Anh Tran Bui, Jatuporn Rattanasrisomporn

**Affiliations:** 1 Faculty of Veterinary Medicine, Vietnam National University of Agricuture, Hanoi 131000, Vietnam; dvhieuvet@vnua.edu.vn (H.V.D.); tthgiang@vnua.edu.vn (G.T.H.T.); hoangvietnguyen1.vnua@gmail.com (H.V.T.N.); btadao@vnua.edu.vn (D.A.T.B.); 2 Department of Companion Animal Clinical Sciences, Faculty of Veterinary Medicine, Kasetsart University, Bangkok 10900, Thailand; 3 GAIA Pet Clinic Ocean Park, Gia Lam, Hanoi 131000, Vietnam; linhpah@gmail.com; 4 Interdisciplinary of Genetic Engineering and Bioinformatics, Graduate School, Kasetsart University, Bangkok 10900, Thailand; fgraapr@ku.ac.th; 5 Faculty of Veterinary Medicine, Phetchaburi Rajabhat University, Phetchaburi 76000, Thailand

**Keywords:** cats, feline kobuvirus, PCR, Vietnam

## Abstract

Feline kobuvirus (FKoV) has been linked to diarrheal disease in cats and has recently emerged in several Asian countries. This study aimed to investigate the epidemiological and genetic characterization of FKoV in domestic cats in northern Vietnam from 2022 to 2025. In total, 244 fecal samples were obtained from both healthy and sick cats in Hanoi, Hungyen, Bacninh, and Ninhbinh in the north of Vietnam. Of 244 fecal samples examined, eight (3.28%) samples were found to be positive for the FKoV genome by using the conventional PCR method. Viral genomes were identified in both healthy and sick cats. The highest FKoV-positive rate was 12.50% in 6–12-month-old cats, significantly higher than those detected in cats aged 3–6 months and >12 months. Eight positive samples were successfully sequenced and characterized for the partial 3D gene and full-length VP1 genes. Results showed that the level of nucleotide identity of the partial 3D gene and full-length VP1 genes among the FKoV strains in this study ranged from 95.86% to 100% and 94.09% to 99.73%, respectively. Phylogenetic analysis revealed that the eight Vietnamese viral strains obtained in this study formed a novel cluster within FKoV and are genetically related to Chinese FKoV strains. Two positive selections were found in the VP1 protein of the Vietnamese FKoV strains.

## 1. Introduction

According to the International Committee on Taxonomy of Viruses in 2017, the kobuvirus genus consists of six species, designated Aichivirus (AiV) A1–A10, B1–B3, C1–C2, D1–D2, E1, and F1–F2 [1]. Among these, Aichivirus A comprises human AiV and feline, murine, canine, rat, and roller kobuvirus, whereas other AiV B, C, D, E, F include kobuvirus in cattle, ferrets, caprine, sheep, pigs, kagovirus 1 and kagovirus 2, rabbit, and bats, respectively [1]. The broad host range and genetic diversity of kobuviruses suggest their successful adaptation to multiple mammalian species and highlight the importance of continued surveillance to better understand their evolution and epidemiology.

Aichivirus, which belongs to the kobuvirus genus, was first identified in 1989 in a patient with oyster-associated food poisoning following the consumption of oysters in Aichi Province, Japan [2]. Since then, aichiviruses have been detected in a wide range of mammalian hosts worldwide, including humans, cattle, pigs, wild boars, dogs, rabbits, and rodents. [3,4,5,6]. The virus was also detected in patients with enteritis symptoms [5], and healthy cases [7].

Feline kobuvirus (FKoV), a member of the species Aichivirus A4 [1], was detected in domestic cats with diarrheal symptoms in South Korea in 2013 [8]. Similar to other viruses within the kobuvirus genus, FKoV is a small, non-enveloped virus with spherical particles approximately 27–30 nm in diameter [1]. The viral genome consists of a single-stranded RNA of approximately 8.2 to 8.4 kb, containing a single open reading frame (ORF). This ORF codes for a polyprotein. The polyprotein generates three structural proteins (VP0, VP1, VP3) and several non-structural proteins (L, 2A to 2C, 3A to 3D) [9]. Among these proteins, VP1 is a main structural protein related to the antigenicity and pathogenicity of the virus. The 3D gene codes for RNA-dependent RNA polymerase (RdRp), playing an essential role in viral replication in host cells [9,10]. Therefore, the 3D and VP1 gene sequences were employed to genetically characterize FKoV strains [11,12,13].

Since the first report in South Korea, FKoV has been documented in numerous countries worldwide such as the United Kingdom [14], Italy [15], Iran [16], and China [11,17]. In Vietnam, kobuvirus (KoV) infection has been reported in several hosts, including humans, pigs, bats, and rodents. [12,18]. To date, only a single FKoV sequence is available in GenBank, and is characterized based on the VP1 gene sequence from a fecal sample in a cat in the Daklak province in central Vietnam [12], limiting our understanding of the genetic diversity and epidemiology of this virus in the country. This study was carried out to investigate FKoV infection in domestic cats and characterize the viral strains circulating in northern Vietnam, using the partial 3D and full-length VP1 genes.

## 2. Results

### 2.1. Detection of Feline Kobuvirus Genome in the Field Samples Using Conventional PCR

Viral genomes were detected in eight (3.28%, 95% CI: 1.43–6.36) out of 244 field samples collected in four city/provinces in northern Vietnam (Table 1). The FKoV-positive rate was 6.25% (95% CI: 1.31–17.20) in cats from Hungyen province, followed by Bacninh (4.23%, 95% CI: 0.88–11.86) and Hanoi (2.50%, 95% CI: 0.3–8.74). No positive sample was detected in cats from Ninhbinh province in this study (Table 1). The FCoV and FPV genomes were not detected in the FKoV-positive samples.

The FKoV-positive rates according to age, gender, breed, and health status of cats were calculated in this study. The results are indicated in Table 2. The obtained data indicated that the highest positive rate for the FKoV genome detection was 12.5% from cats at 6–12 months old, significantly higher than that of cats at above 12 months old (1.16%, *p* = 0.0014 < 0.01). FKoV was not detected in 3–6-month-old cats in this study. Regarding health status, samples were positive for the FKoV genome in healthy, diarrheal, and non-diarrheal cats with significant positive rates of 5.26%, 13.64%, and 0.68% (*p* = 0.0008), respectively. In contrast, positive rates did not differ significantly with breed (*p* = 0.46) and gender (*p* = 0.95) of the obtained cats (Table 2).

### 2.2. Genetic and Phylogenetic Analysis of the Feline Kobuvirus Strains

#### 2.2.1. Genetic and Phylogenetic Characterization of the Partial 3D Gene Sequences of the Vietnamese Feline Kobuvirus Strains

Eight positive samples were successfully sequenced and designated as follows: Feline/Vietnam/FKoV/VNUA-96, -116, -119, -121, -175, -189, -201, and -202. The analysis of nucleotide identity of the partial 3D gene sequences among Vietnamese FKoV strains indicated that the nucleotide identity was from 95.86% (VNUA-96 vs. VNUA-116) to 100% (VNUA-121 vs. VNUA-116) (Table 3). In comparison to that of the one Vietnamese sequence in GenBank, its nucleotide identity was from 92.97% (Vietnam/16915x11/2013 vs. VNUA-96) to 94.62% (Vietnam/16915x11/2013 vs. VNUA-189) (Table 3). To compare with those available in GenBank, the highest nucleotide identity was from 93.75% (VNUA-119 vs. Feline/China/17HRB0903/2017 (17HRB0903)) to 95.53% (VNUA-189 vs. Feline/China/17HRB0903/2017 (17HRB0903)) (Table 4).

**Figure 1:**
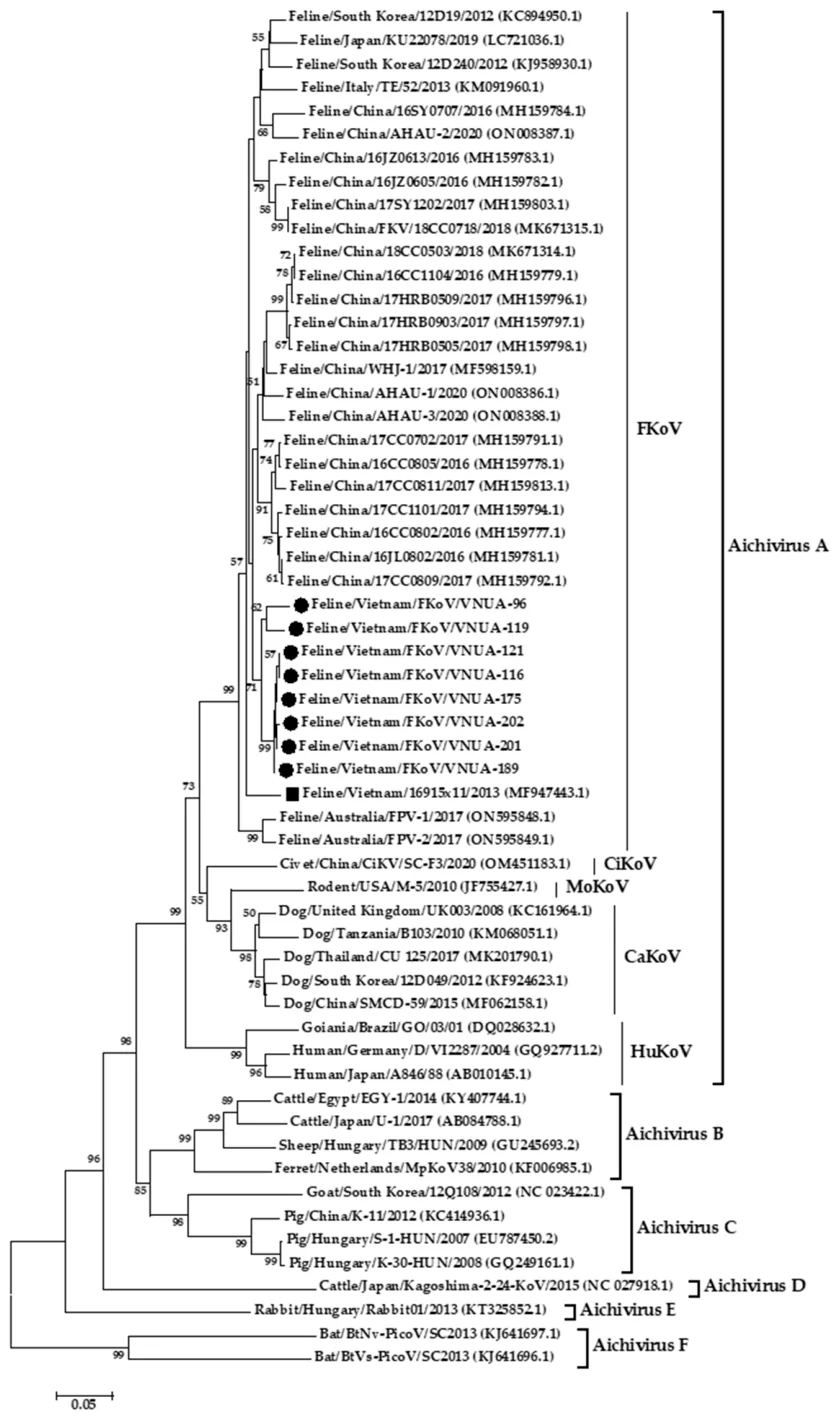
Phylogenetic tree of the partial 3D gene sequences (484 bp) of the Vietnamese FKoV strains and sequences downloaded from Genbank database. The Vietnamese FKoV strains from the present study are indicated by black circles, while the previously reported Vietnamese strain is indicated by a black square.

The establishment of a phylogenetic tree was performed using the partial 3D gene sequences of eight Vietnamese FKoV strains and other sequences from GenBank. Figure 1 indicated that eight Vietnamese FKoV formed a single cluster and belonged to FKoV within the Aichivirus A species. The Vietnamese FKoV strains were genetically close to Chinese viral strains (Figure 1).

#### 2.2.2. Genetic and Phylogenetic Analysis of the Complete VP1 Gene Sequences of the Vietnamese Feline Kobuvirus Strains

Analysis of nucleotide identity among the eight FKoV strains obtained indicated that the nucleotide identity was from 94.09% (VNUA-96 vs. VNUA-121, VNUA-119 vs. VNUA-121) to 99.73% (VNUA-121 vs. VNUA-175, VNUA-121 vs. VNUA-189, VNUA-175 vs. VNUA-189). To compare that of previous Vietnamese FKoV strains, their nucleotide identity ranged from 93.7% (VNUA-121 vs. Vietnam/16915x11/2013) to 95.14% (VNUA-96 vs. Vietnam/16915x11/2013) (Table 5). The deduced amino acid identity ranged from 99.90% (VNUA-96 vs. VNUA-119; VNUA-96 vs. VNUA-121; VNUA-119 vs. VNUA-121, VNUA-175, VNUA-189, VNUA-201) to 99.99% (VNUA-116 vs. VNUA-119) (Table 5).

Eight FKoV strains obtained in this study were used to compare nucleotide identity with reference strains previously published in the GenBank based on the full-length VP1 gene sequences. The highest nucleotide identity was found in Chinese strains (Feline/China/16JL0802/2016 (MK671282.1) and Feline/China/WHJ-1/2017 (MF598159.1)), ranging from 94.09% to 95.93% (Table 6).

**Figure 2:**
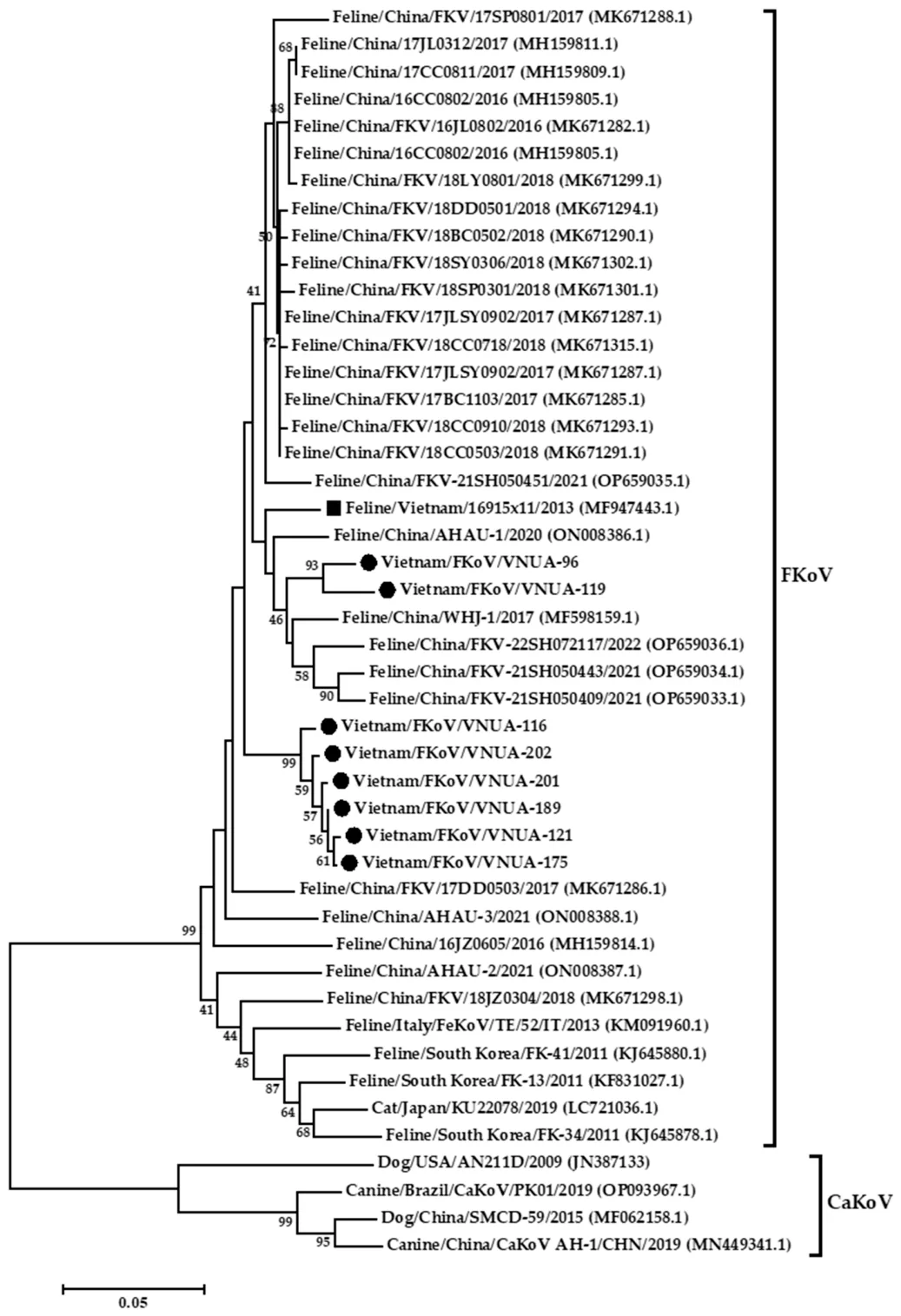
Phylogenetic tree of the complete VP1 gene sequences of the Vietnamese feline kobuvirus and sequences retrieved from the Genbank database. The Vietnamese FKoV strains from the present study are indicated by black circles, while the previously reported Vietnamese strain is indicated by a black square.

Phylogenetic analysis of the eight FKoV strains in northern Vietnam using the full-length VP1 genes showed that the eight sequences in this study belong to two genetic subclusters within FKoV (Figure 2). Two FKoV strains (VNUA-96 and VNUA-119) and a previous Vietnamese Feline/Vietnam/16915x11/2013 (MF947443.1) strain were clustered with the Chinese strain (Feline/China/WHJ-1/2017 (MF598159.1)). The remaining six Vietnamese FKoV strains formed a single genetic cluster and are closely related to Chinese viral strains (Figure 2).

Substitutions in deduced amino acids were identified in the VP1 protein among Vietnamese FKoV strains. The data are indicated in Table 7. Results showed that five substitutions were detected among Vietnamese FKoV strains, including sites 58 (T → A), 62 (V → I), 83 (T → V), 250 (I → L), and 252 (K → R). Substitutions at sites 58-A (VNUA-121 and VNUA-175) and 83-V (VNUA-116, -121, -175, -189, -201, and -202) are unique in Vietnamese FKoV strains (Table 7).

### 2.3. Evolutionary Analysis of the Full-Length VP1 Gene Sequence of the Vietnamese Feline Kobuvirus Strains

#### 2.3.1. Analysis of Recombination Events of Vietnamese FKoV Strains

Analysis of recombination was performed using the full-length VP1 gene sequences from eight FKoV strains, and that of other reference strains in Genbank was done using RDP version 5.0 software. No genetic recombination events were identified among FKoV strains in northern Vietnam.

#### 2.3.2. Natural Selection Analysis of the Vietnamese FKoV Strains

From the analysis of the Vietnamese FKoV VP1 protein sequences (256 amino acids) obtained, two sites under positive selection were determined in the VP1 protein, which were located at sites 250 and 252 (Table 8). Conversely, 106 negative selection sites were identified in the VP1 protein (Supplementary Table S1).

## 3. Discussion

FKoV infection was first reported in South Korea in 2013 [8]. Since then, viral infection has been reported in sick and healthy cats in the world [11,13,15,16,17]. In Vietnam, there was only one sequence of FKoV available in GenBank to date [12]. This is, to our knowledge, the second documentation of FKoV infection and genetic characterization in domestic cats in Vietnam. A low FKoV-positive rate (3.28%) was observed in fecal samples from cats in four regions in the north of Vietnam. Genetic and phylogenetic analysis based on the partial 3D gene (484 bp) and VP1 gene (759 bp) revealed that the eight FKoV strains obtained in this study exhibited high nucleotide identity, ranging from 95.86% to 100% and 94.09% to 99.73%, respectively, which formed a single cluster and are genetically related to FKoV strains from China. Two positive selections were found in the VP1 protein among Vietnamese FKoV strains.

Regarding FKoV infection, the positive rates vary among countries in the world. In this study, the positivity rate of FKoV infection was 3.28%, which is significantly lower than the 9.9% to 14.2% reported in China [11,19], 15.4% in South Korea [8], and 13.5% in Italy [15]. These different rates were due to the fact that different sampling regions, sample types, health status, and detection methods were used to identify the viral genome in field samples. Additional studies should be conducted to expand sample size and apply a highly sensitive detection method to give an overlook of FKoV infection across Vietnam. Interestingly, the FKoV infection based on the partial 3D gene could be identified in samples collected from both cats with diarrheal symptoms and cats of healthy status [8,11,15,19]. Similarly to this study, we also detected FKoV in diarrheal cats (13.64%) and healthy cats (5.26%). The eight FKoV-positive samples identified in this study were not co-infected with the common feline coronavirus or feline panleukopenia virus. Further investigation of other viral (feline astrovirus, rotavirus, etc.) and bacterial (*Escherichia coli*, *Salmonella* spp., *Giardia* spp., etc.) pathogens in FKoV-positive samples is warranted to determine whether co-infections contribute to the development of diarrheal signs in cats. In addition, Chung et al. found that FKoV genome was detected in six cats at below six months of age [8]. The highest FKoV-positive rate was detected in 6–12-month-old cats (Table 2) in this study.

Based on phylogenetic evaluation of the partial 3D genes, it was previously noted that Chinese FKoV strains exhibit a unique evolutionary trend [11]. In this study, phylogenetic evaluation of the partial 3D genes and complete VP1 genes revealed that Vietnamese viral strains formed a novel and single cluster. These findings support the possibility that a distinct FKoV cluster may have evolved within Vietnam.

The VP1 protein plays a crucial role in determining the antigenic properties and pathogenic potential of FKoV strains, making it an important target for the characterization of viruses in molecular studies. In addition, this protein is variable compared to other structural proteins of KoV strains [9]. Niu et al. [11] identified six amino acid substitutions in the VP1 protein of Chinese FKoV strains at positions 22 (N/Y), 108 (I/T), 149 (T/A), 182 (T/A), 186 (A/V), and 235 (P/S). In the VP1 protein, a polyproline-rich fragment (position 227 to 243) is associated with antigen recognition, signal transduction, viral infectivity and pathogenicity [20]. A substitution was identified within the polyproline-rich fragment spanning positions 227 to 243, specifically at position 235, where proline was replaced by serine (P → S). The author suggested that this substitution may affect viral activity [11]. In the present study, the polyproline-rich fragment was highly conserved among Vietnamese FKoV strains. Further studies are planned to expand upon this point. In addition, two positive selections were first reported in this study at sites 250 and 252 of the VP1 protein (Table 8), with the details of amino acids indicated in Table 7. To date, there is no evidence indicating any biological or virulence changes related to these amino acid substitutions.

Recombination is one of the evolutionary processes which have been reported among KoV strains to generate novel viral strains or genotypes [21,22]. Deng et al. reported that recombination events occurred between feline kobuvirus and canine kobuvirus to generate a novel viral strain in stray dogs [23]. In this study, we did not detect recombination events between the Vietnamese FKoV strains using the full-length VP1 gene sequences. The complete genomes of the viral strains should be sequenced and analyzed to further clarify the appearance of recombination events among FKoV in Vietnam.

Several limitations should be acknowledged. The relatively small sample size and limited geographic distribution of the samples may restrict the broader applicability of the findings. Furthermore, the genetic and evolutionary analyses were conducted using partial sequences of the 3D and VP1 genes, which may not fully reflect the complete evolutionary dynamics of FKoV circulating in Vietnam. Future research should expand the sample size and geographic coverage, as well as perform whole-genome sequencing and viral isolation to enable more comprehensive analyses of the biological, genetic characteristics and evolutionary dynamics of the virus across Vietnam.

## 4. Materials and Methods

### 4.1. Ethics Statement

All procedures involving the handling of animals and the collection of biological samples were conducted in strict compliance with the ethical guidelines established by the Vietnam National University of Agriculture under approval number CARE-2022/08. The study was approved by the University’s Committee on Animal Research and Ethics. Written informed consent was obtained from all cat owners, and sampling was performed to minimize stress, following international animal welfare standards.

### 4.2. Performance of Sample Collection

In total, 244 fecal samples were taken from domestic cats aged 3 to 36 months from various veterinary clinics in Hanoi City and the Hungyen, Bacninh, and Ninhbinh provinces in northern Vietnam between 2022 and 2025. Some cats visited the veterinary clinics with clinical signs such as fever, vomiting, diarrhea, and others; other cats came to the veterinary clinics with a healthy status. Samples were placed in 1 mL of phosphate-buffered saline (PBS) and stored at 2 °C to 8 °C, and transported to the Faculty of Veterinary Medicine, Vietnam National University of Agriculture. Samples were homogenized by thorough vortexing and centrifuged at 1500 rpm for 5 min. The collected supernatant was preserved at −80 °C until analyzed.

### 4.3. Total RNA Extraction, cDNA Synthesis, and PCR for Viral Genome Detection

The commercial Viral Gene-spin™ Viral DNA/RNA Extraction Kits (Intron, Seoul, Korea) were employed to extract total RNA. Following the protocol provided by the manufacturer, the procedure was performed. Synthesis of cDNA was performed using reverse transcriptase M-MLV (Promega, Madison, WI, USA) with a random primer. The mixture was incubated in a thermal cycler as 37 °C for 60 min, followed by 72 °C for 5 min.

Conventional PCR was performed using FKoV-3D-F/R, FPV-F/R, and FCoV-P205/P211 primers to amplify a partial gene sequence for FKoV, feline panleukopenia virus (FPV), and feline coronavirus (FCoV) detection in field samples, respectively, as previously described [11,24,25]. A 25 µL mixture, including 12.5 µL of GoTaq^TM^ Green Master Mix (Promega), 1 µL of each forward/reverse primer (10 mM) (Table 9) [11], 8.5 µL of nuclease-free water, and 2 µL of cDNA template, was used. The PCR conditions were set as an initial denaturation step at 94 °C for 5 min, with 35 cycles (94 °C for 60 s, 62 °C (for FKoV), 60 °C (for FPV), 55 °C (for FCoV) for 45 s, 72 °C for 60 s) and a final extension at 72 °C for 10 min. PCR products were analyzed by electrophoresis on a 1.5% agarose gel. PCR products were visualized using an ultraviolet transilluminator.

### 4.4. Performance of PCR for Amplifying the Full-Length VP1 Gene Sequence

The positive samples were sent for sequencing of the partial 3D and full-length VP1 genes using FKoV-3D-F/FKoV-3D-R and FKoV-VP1-F/FKoV-VP1-R, respectively (Table 9) [11]. A 25 μL mixture for PCR amplification was mentioned above and set as an initial denaturation step at 95 °C for 5 min, with 35 cycles (95 °C for 30 s, 60 °C (VP1 gene amplification) for 45 s, 72 °C for 30 s) and a final extension at 72 °C for 10 min.

### 4.5. Nucleotide Sequencing

PCR products were transferred to 1st BASE (Selangor, Malaysia) for Sanger sequencing. The partial 3D and full-length VP1 genes sequences were deposited into GenBank under accession numbers: PZ276909 to PZ276924.

### 4.6. Genetic and Phylogenetic Analyses

The Cluster W [26] in the BioEdit software version 7.2 [27] was used to align nucleotide sequences in this study, and along with additional sequences retrieved from GenBank (Table 10). Calculation of nucleotide identity was performed using GENETYX version 10.0 (GENETYX Corp., Tokyo, Japan) and the Basic Local Alignment Search Tool (BLAST) program (https://blast.ncbi.nlm.nih.gov/).

The partial 3D genes and full-length VP1 gene sequences of FKoV obtained were used to construct phylogenetic trees by using MEGA software version 7.0 (https://megasoftware.net/). The maximum-likelihood method was used to construct phylogenetic trees (1000 replicates).

### 4.7. Analysis of Recombination Events and Natural Selection Profile

Recombination Detection Program (RDP) version 5.0 [28] was applied to detect potential recombination events among the FKoV strains. RDP, BootScan, Chimaera, MaxChi, GENECONV, SiScan, LARD, 3Seq, and PhylPro algorithms were used to examine recombination events. The program was run with default parameters. A significance threshold of *p* < 0.05 was used to determine recombination.

Performance of Datamonkey (http://www.datamonkey.org/) was done by following the Fast Unconstrained Bayesian AppRoximation (FUBAR) method [29] to detect the estimation of evolutionary selection profiles. Model selection tests identified the general time-reversible model as the best fit for the evolutionary data.

### 4.8. Statistical Analysis

Fisher’s exact test assessed associations between infection status and host variables, with statistical significance defined at *p* < 0.05.

## 5. Conclusions

FKoV infection was detected in eight (3.28%) out of 244 samples in domestic cats from four regions in northern Vietnam during 2022 to 2025. Viral infection was detected in healthy and sick cats in this study. The positivity rate was 12.50% among cats aged 6–12 months, which was markedly higher than the value observed in cats aged 3–6 months and >12 months. The eight partial 3D gene and full-length VP1 gene sequences of Vietnamese FKoV were successfully sequenced and characterized. The nucleotide identity of the partial 3D gene and VP1 genes among the FKoV strains ranged from 95.86% to 100% and 94.09% to 99.73%, respectively. Phylogenetic trees revealed that the eight Vietnamese viral strains formed a novel cluster within FKoV and are genetically related to Chinese FKoV strains. Two positive selections were found in the VP1 protein of the Vietnamese FKoV.

## Figures and Tables

**Table 1:** Identification of the feline kobuvirus genome in field samples.

No.	Province/City	No. of Tested Samples	No. of Gene-Positive Samples	Positive Rate (%)	95% CI
1	Hanoi	80	2	2.50	0.3–8.74
2	Bacninh	71	3	4.23	0.88–11.86
3	Hungyen	48	3	6.25	1.31–17.20
4	Ninhbinh	45	0	0	-
Total		244	8	3.28	1.43–6.36

**Table 2:** Identification of the feline kobuvirus genome among field samples based on age, gender, breed, and health status.

Criteria		No. of Tested Samples	No. of Gene-Positive Samples	Positive Rate (%)	95% CI	*p*-Value
Breed	Native cats	40	2	5.00	0.61–16.92	0.46
	Exotic, cross-breed cats	204	6	2.94	1.09–6.29	
Age (Months)	3–6	24	0	0	-	0.0014
	6–12	48	6	12.50	4.73–25.25	
	>12	172	2	1.16	0.14–4.14	
Gender	Male	125	4	3.20	0.88–7.99	0.95
	Female	119	4	3.36	0.92–8.38	
Health status	Healthy	76	4	5.26	1.45–12.93	0.0008
	Diarrhea	22	3	13.64	2.91–34.91	
	Non-diarrhea	146	1	0.68	0.02–3.76	

**Table 3:** Analysis of nucleotide identity in the partial 3D gene sequences (484 bp) of Vietnamese FKoV strains.

Strain Name	Nucleotide Identity (%)							
	VNUA-96	VNUA-116	VNUA-119	VNUA-121	VNUA-175	VNUA-189	VNUA-201	VNUA-202
VNUA-96	100							
VNUA-116	95.86	100						
VNUA-119	96.28	96.9	100					
VNUA-121	95.86	100	96.9	100				
VNUA-175	96.07	99.79	97.1	99.79	100			
VNUA-189	96.28	99.58	97.31	99.58	99.79	100		
VNUA-201	96.28	99.58	96.9	99.58	99.38	99.58	100	
VNUA-202	96.28	99.58	96.9	99.58	99.38	99.58	99.58	100
Vietnam/16915x11/2013	92.97	94.21	94.42	94.21	94.42	94.62	94.21	94.21

**Table 4:** Detection of the highest nucleotide identity of the partial 3D gene sequences (484 bp) of Vietnamese FKoV strains in this study with those of others from GenBank.

Strain Name	Virus with the Highest Nucleotide Identity					
	GenBank Accession Number	Strain Name	Source	Country	Year	%
VNUA-96	MH159797.1	17HRB0903	Cat	China	2017	94.41
VNUA-116	MH159797.1	17HRB0903	Cat	China	2017	95.08
VNUA-119	MH159797.1	17HRB0903	Cat	China	2017	93.75
VNUA-121	MH159797.1	17HRB0903	Cat	China	2017	95.08
VNUA-175	MH159797.1	17HRB0903	Cat	China	2017	95.31
VNUA-189	MH159797.1	17HRB0903	Cat	China	2017	95.53
VNUA-201	MH159797.1	17HRB0903	Cat	China	2017	95.08
VNUA-202	MH159797.1	17HRB0903	Cat	China	2017	95.08

**Table 5:** Analysis of nucleotide identity of the Vietnamese feline kobuvirus in this study based on VP1 genes (759 bp).

Strain Name	VNUA-96	VNUA-116	VNUA-119	VNUA-121	VNUA-175	VNUA-189	VNUA-201	VNUA-202	
VNUA-96	100	99.96	99.90	99.90	99.91	99.91	99.91	99.95	Amino Acid Identity (%)
VNUA-116	95.01	100	99.99	99.98	99.99	99.99	99.98	99.93	
VNUA-119	97.37	95.01	100	99.90	99.90	99.90	99.90	99.94	
VNUA-121	94.09	98.81	94.09	100	99.99	99.99	99.99	99.92	
VNUA-175	94.35	98.81	94.35	99.73	100	99.99	99.98	99.92	
VNUA-189	94.35	98.81	94.35	99.73	99.73	100	99.99	99.93	
VNUA-201	94.48	98.95	94.22	99.6	99.34	99.6	100	99.93	
VNUA-202	94.61	98.55	94.61	99.21	99.21	99.21	99.34	100	
Vietnam/16915x11/2013	95.14	94.48	94.88	93.7	93.96	93.96	93.96	94.22	
	Nucleotide Identity (%)								

**Table 6:** Detection of the highest nucleotide identity of the Vietnamese feline kobuvirus and others from GenBank using the VP1 genes.

Strain Name	Virus with the Highest Nucleotide Identity					
	GenBank Accession Number	Strain Name	Source	Country	Year	%
VNUA-96	MF598159.1	WHJ-1	Cat	China	2017	95.93
VNUA-116	MK671282.1	16JL0802	Cat	China	2016	94.61
VNUA-119	MF598159.1	WHJ-1	Cat	China	2017	95.80
VNUA-121	MK671282.1	16JL0802	Cat	China	2016	94.09
VNUA-175	MK671282.1	16JL0802	Cat	China	2016	94.09
VNUA-189	MK671282.1	16JL0802	Cat	China	2016	94.35
VNUA-201	MK671282.1	16JL0802	Cat	China	2016	94.35
VNUA-202	MK671282.1	16JL0802	Cat	China	2016	94.61

**Table 7:** Substitutions of amino acids in the VP1 protein of the Vietnamese FKoV strains.

Virus Strain	Amino Acid Substitutions on VP1 Protein				
	58	62	83	250	252
Reference Strains:					
China/16CC0802/2016_(MH159805.1)	T	V	T	I	K
Italy/FeKoV/TE/52/IT/2013 (KM091960.1)					
South Korea/FK-13/2011 (KF831027.1)				V	
Japan/KU22078/2019 (LC721036.1)					R
Vietnam/16915x11/2013 (MF947443.1)				L	
Vietnamese Strains in This Study:					
VNUA-96					
VNUA-116			V		
VNUA-119		I		L	R
VNUA-121	A		V	L	
VNUA-175	A		V	L	
VNUA-189			V	L	
VNUA-201			V		
VNUA-202			V	L	

**Table 8:** Assessment of sites under positive selection of VP1 protein sequences of the Vietnamese feline kobuvirus strains.

No.	Site	a	b	a–b	Prob [a > b]	Prob [a < b]	Bayes Factor [a < b]
1	250	0.56	7.36	6.80	0.00	0.99	298.20
2	252	0.54	6.55	6.01	0.00	0.99	186.52

**Table 9:** Primer sequences used in this study.

No.	Primer Name	Sequence (5′-3′)	PCR Product (bp)	References
1	FKoV-3D-F	CTC CGC CCC ACC GCT AAG G	530	[11]
2	FKoV-3D-R	GGG GGT TCC GTT GCG TAG ATG A		
3	FKoV-VP1-F	GCC CCC GCC TCT GCC ATT GTG	843	
4	FKoV-VP1-R	CTT GAT GAC GGC GAC GGA CTT TTC		
5	FCoV-P205	GGC AAC CCG ATG TTT AAA ACT GG	223	[25]
6	FCoV-P211	CAC TAG ATC CAG ACG TTA GCT C		
7	FPV-F	TGCCTCAATCTGAAGGAGCT	250	[24]
8	FPV-R	TTTCATCTGTTTGCGCTCCC		

**Table 10:** The 3D and VP1 gene sequences of kobuvirus used in this study.

No.	GenBank Accession Number	Strain Name	Country	Source	Year	Gene Sequence	
						3D	VP1
1	KC414936.1	K-11	China	Pig	2012	X	
2	KJ641697.1	BtNv-PicoV/SC2013	China	Bat	2013	X	
3	KJ641696.1	BtVs-PicoV/SC2013	China	Bat	2013	X	
4	MF062158.1	SMCD-59	China	Dog	2015	X	X
5	MH159783.1	16JZ0613	China	Cat	2016	X	
6	MH159784.1	16SY0707	China	Cat	2016	X	
7	MH159777.1	16CC0802	China	Cat	2016	X	X
8	MH159779.1	16CC1104	China	Cat	2016	X	
9	MH159778.1	16CC0805	China	Cat	2016	X	
10	MH159781.1	16JL0802	China	Cat	2016	X	X
11	MH159811.1	17JL0312	China	Cat	2017		X
12	MK671286.1	17DD0503	China	Cat	2017		X
13	MK671285.1	17BC1103	China	Cat	2017		X
14	MK671288.1	17SP0801	China	Cat	2017		X
15	MK671287.1	17JLSY0902	China	Cat	2017		X
16	MF598159.1	WHJ-1	China	Cat	2017	X	X
17	MH159813.1	17CC0811	China	Cat	2017	X	X
18	MH159797.1	17HRB0903	China	Cat	2017	X	
19	MH159798.1	17HRB0505	China	Cat	2017	X	
20	MH159796.1	17HRB0509	China	Cat	2017	X	
21	MH159791.1	17CC0702	China	Cat	2017	X	
22	MH159794.1	17CC1101	China	Cat	2017	X	
23	MH159792.1	17CC0809	China	Cat	2017	X	
24	MH159803.1	17SY1202	China	Cat	2017	X	
25	MH159782.1	16JZ0605	China	Cat	2017	X	X
26	MK671298.1	18JZ0304	China	Cat	2018		X
27	MK671290.1	18BC0502	China	Cat	2018		X
28	MK671314.1	18CC0503	China	Cat	2018	X	X
29	MK671315.1	18CC0718	China	Cat	2018	X	X
30	MK671293.1	18CC0910	China	Cat	2018		X
31	MK671302.1	18SY0306	China	Cat	2018		X
32	MK671294.1	18DD0501	China	Cat	2018		X
33	MK671299.1	18LY0801	China	Cat	2018		X
34	MK671301.1	18SP0301	China	Cat	2018		X
35	MN449341.1	CHN	China	Dog	2019		X
36	ON008386.1	AHAU-1	China	Cat	2020	X	X
37	MH159813.1	AHAU-2	China	Cat	2020	X	X
38	OM451183.1	CiKV/SC-F3	China	Civet	2020	X	
39	OP659034.1	FKV-21SH050443	China	Cat	2021		X
40	ON008388.1	AHAU-3	China	Cat	2021		X
41	OP659033.1	FKV-21SH050409	China	Cat	2021		X
42	OP659035.1	FKV-21SH050451	China	Cat	2021		X
43	OP659036.1	FKV-22SH072117	China	Cat	2022		X
44	MF947443.1	16915x11	Vietnam	Cat	2017	X	X
45	KF831027.1	FK-13	South Korea	Cat	2011		X
46	KJ645878.1	FK-34	South Korea	Cat	2011		X
47	KJ645880.1	FK-41	South Korea	Cat	2011		X
48	KC894950.1	12D19	South Korea	Cat	2012	X	
49	KF924623.1	12D049	South Korea	Dog	2012	X	
50	NC_023422.1	12Q108	South Korea	Goat	2012	X	
51	KJ958930.1	12D240	South Korea	Cat	2012	X	
52	AB010145.1	A846	Japan	Human	1988	X	
53	NC_027918.1	Kagoshima-2-24	Japan	Cattle	2015	X	
54	KC161964.1	U-1	Japan	Cattle	2017	X	
55	LC721036.1	KU22078	Japan	Cat	2019	X	X
56	ON595848.1	FPV-1	Australia	Cat	2017	X	
57	ON595849.1	FPV-2	Australia	Cat	2017	X	
58	KM091960.1	TE/52	Italy	Cat	2013	X	X
59	KY407744.1	EGY-1	Egypt	Cattle	2014	X	
60	KC161964.1	UK003	UK	Dog	2008	X	
61	MK201790.1	CU_125	Thailand	Dog	2017	X	
62	KM068051.1	B103	Tanzania	Dog	2010	X	
63	KF006985.1	MpKoV38	Netherlands	Ferret	2010	X	
64	DQ028632.1	GO/03	Brazil	Goiania	2001	X	
65	OP093967.1	PK01	Brazil	Dog	2019		X
66	GQ927711.2	D/VI2287	Germany	Human	2004	X	
67	JN387133	AN211D	USA	Dog	2009		X
68	JF755427.1	M-5	USA	Rodent	2010	X	
69	EU787450.2	S-1-HUN	Hungary	Pig	2007	X	
70	GQ249161.1	K-30-HUN	Hungary	Pig	2008	X	
71	GU245693.2	TB3/HUN	Hungary	Sheep	2009	X	
72	KT325852.1	Rabbit01	Hungary	Rabbit	2013	X	

## Data Availability

The data presented in this study are available within the article. Raw data supporting this study are available from the corresponding author.

## Supplementary Materials

The following supporting information can be downloaded at: https://www.mdpi.com/article/10.3390/ijms27167394/s1.
