# Supplementary material for: Feline Kobuvirus in Domestic Cats in Northern Vietnam: Molecular Insights Reveal Adaptive Evolution in VP1 Protein
Source: Int J Mol Sci. 2026 Aug 18;27(16):7394. doi: 10.3390/ijms27167394 (PMC13512943; doi:10.3390/ijms27167394)
Supplement: Supplementary file 1 [file ijms-27-07394-s001.zip › ijms-4407483-supplementary.pdf]

Supplementary Table S1. Analysis of negative selection profile of VP1 protein sequences of the Vietnamese feline kobuvirus strains.

No.	Site	a	b	a–b	Prob [a>b]	Prob [a<b]	Bayes Factor [a<b]
1	1	3.14	0.12	-3.02	0.99	0.01	0.02
2	4	19.57	0.44	-19.14	1.00	0.00	0.00
3	5	2.82	0.15	-2.67	0.99	0.01	0.01
4	8	7.19	0.17	-7.02	1.00	0.00	0.00
5	9	5.99	0.19	-5.80	0.99	0.01	0.02
6	10	4.08	0.18	-3.90	0.96	0.03	0.06
7	12	4.00	0.15	-3.85	0.99	0.01	0.02
8	13	6.51	0.18	-6.34	0.99	0.01	0.02
9	17	2.34	0.14	-2.19	0.96	0.03	0.07
10	18	2.62	0.11	-2.51	0.99	0.01	0.01
11	22	2.34	0.14	-2.19	0.96	0.03	0.07
12	23	10.05	0.41	-9.64	1.00	0.00	0.00
13	26	2.38	0.16	-2.21	0.95	0.04	0.08
14	27	9.95	0.13	-9.81	1.00	0.00	0.00
15	29	2.62	0.16	-2.46	0.95	0.04	0.08
16	30	2.46	0.08	-2.38	0.98	0.02	0.04
17	35	4.79	0.13	-4.65	0.99	0.01	0.01
18	37	9.56	0.14	-9.42	1.00	0.00	0.00
19	38	5.95	0.18	-5.77	1.00	0.00	0.00
20	41	2.10	0.15	-1.95	0.95	0.04	0.09
21	42	2.18	0.14	-2.04	0.96	0.03	0.07
22	47	3.47	0.07	-3.40	0.99	0.00	0.01
23	48	6.41	0.14	-6.27	1.00	0.00	0.00
24	49	4.30	0.07	-4.23	1.00	0.00	0.01
25	50	2.65	0.13	-2.52	0.97	0.03	0.06
26	52	2.14	0.14	-2.00	0.95	0.04	0.08
27	53	3.89	0.13	-3.75	0.99	0.01	0.02
28	56	5.17	0.13	-5.04	1.00	0.00	0.00
29	57	12.98	0.17	-12.81	1.00	0.00	0.00
30	59	4.22	0.30	-3.93	0.98	0.02	0.04
31	60	2.90	0.26	-2.64	0.97	0.02	0.04
32	61	7.23	0.49	-6.74	0.99	0.01	0.02
33	64	2.05	0.33	-1.72	0.94	0.05	0.10
34	66	4.92	0.56	-4.36	0.96	0.03	0.07
35	67	15.65	0.35	-15.30	1.00	0.00	0.00
36	68	18.91	0.56	-18.36	1.00	0.00	0.00
37	69	4.84	0.35	-4.49	0.98	0.02	0.04
38	70	29.90	0.41	-29.49	1.00	0.00	0.00
39	71	6.54	0.33	-6.20	0.92	0.07	0.15

40	73	7.42	0.35	-7.08	1.00	0.00	0.01
41	74	5.33	0.34	-5.00	0.99	0.00	0.01
42	75	2.57	0.34	-2.23	0.95	0.04	0.09
43	76	1.64	0.36	-1.28	0.90	0.07	0.17
44	80	4.74	0.74	-4.00	0.92	0.05	0.12
45	81	1.43	0.18	-1.25	0.91	0.08	0.17
46	82	3.86	0.53	-3.33	0.91	0.07	0.15
47	84	5.43	0.61	-4.82	0.93	0.05	0.12
48	87	2.76	0.63	-2.13	0.92	0.06	0.13
49	89	1.69	0.19	-1.50	0.95	0.04	0.08
50	102	3.48	0.44	-3.04	0.99	0.01	0.02
51	105	4.22	0.49	-3.73	0.94	0.05	0.10
52	112	1.71	0.38	-1.32	0.90	0.07	0.17
53	113	4.09	0.46	-3.62	0.99	0.01	0.01
54	117	3.22	0.36	-2.86	0.97	0.02	0.04
55	119	3.03	0.50	-2.53	0.94	0.05	0.11
56	120	3.49	0.34	-3.16	0.99	0.01	0.01
57	123	3.49	0.50	-3.00	0.97	0.02	0.04
58	124	3.24	0.19	-3.05	0.97	0.02	0.05
59	127	3.31	0.34	-2.97	0.99	0.01	0.02
60	129	5.46	0.32	-5.14	1.00	0.00	0.00
61	132	9.57	0.84	-8.73	0.98	0.01	0.03
62	138	3.22	0.35	-2.88	0.96	0.03	0.06
63	140	4.82	0.58	-4.24	0.93	0.05	0.12
64	142	5.02	0.44	-4.58	0.98	0.02	0.04
65	146	5.49	0.49	-5.00	0.95	0.03	0.08
66	147	3.57	0.35	-3.22	0.96	0.03	0.06
67	150	2.81	0.49	-2.32	0.95	0.04	0.08
68	151	3.82	0.57	-3.25	0.91	0.07	0.15
69	154	3.13	0.48	-2.65	0.96	0.03	0.07
70	156	8.76	0.48	-8.28	1.00	0.00	0.00
71	157	3.85	0.30	-3.55	1.00	0.00	0.01
72	158	2.54	0.30	-2.24	0.91	0.07	0.15
73	160	10.44	0.47	-9.97	1.00	0.00	0.00
74	162	6.75	0.60	-6.15	0.94	0.05	0.11
75	163	2.93	0.45	-2.48	0.96	0.03	0.07
76	164	3.86	0.34	-3.52	0.97	0.02	0.05
77	165	1.80	0.36	-1.44	0.91	0.07	0.15
78	181	6.63	0.49	-6.14	0.99	0.01	0.02
79	185	3.62	0.46	-3.16	0.98	0.01	0.02
80	186	4.87	0.80	-4.07	0.93	0.04	0.09
81	190	6.98	0.63	-6.36	0.98	0.01	0.03
82	193	3.76	0.63	-3.12	0.92	0.06	0.13
83	195	4.90	0.13	-4.77	1.00	0.00	0.00
84	196	2.21	0.14	-2.07	0.96	0.03	0.07

85	200	7.60	0.47	-7.13	0.99	0.01	0.02
86	204	11.25	0.48	-10.77	0.99	0.01	0.01
87	206	4.19	0.50	-3.69	0.92	0.06	0.15
88	208	11.96	0.49	-11.47	1.00	0.00	0.00
89	211	4.31	0.36	-3.96	1.00	0.00	0.00
90	213	4.32	0.35	-3.97	0.97	0.02	0.05
91	217	8.91	0.31	-8.60	1.00	0.00	0.00
92	218	3.69	0.50	-3.19	0.96	0.03	0.07
93	223	11.46	0.69	-10.77	1.00	0.00	0.00
94	226	4.25	0.47	-3.78	0.99	0.01	0.01
95	227	6.64	0.50	-6.14	0.99	0.01	0.02
96	228	33.86	0.58	-33.28	1.00	0.00	0.00
97	229	4.05	0.18	-3.87	0.99	0.01	0.02
98	230	2.34	0.19	-2.15	0.96	0.03	0.07
99	231	2.02	0.19	-1.83	0.96	0.03	0.07
100	232	2.58	0.34	-2.24	0.97	0.02	0.05
101	233	8.05	0.32	-7.73	1.00	0.00	0.00
102	236	4.60	0.32	-4.28	0.98	0.02	0.03
103	237	5.27	0.50	-4.77	0.95	0.04	0.09
104	238	7.58	0.65	-6.92	0.99	0.01	0.02
105	240	3.62	0.48	-3.14	0.96	0.03	0.07
106	253	4.53	0.39	-4.14	0.97	0.02	0.05
